# Policy analysis for deciding on a malaria vaccine RTS,S in Tanzania

**DOI:** 10.1186/s12936-016-1197-6

**Published:** 2016-03-08

**Authors:** Idda Romore, Ritha J. A. Njau, Innocent Semali, Aziza Mwisongo, Antoinette Ba Nguz, Hassan Mshinda, Marcel Tanner, Salim Abdulla

**Affiliations:** Swiss Tropical and Public Health Institute, Socinstrasse 57, Postfach, 4002 Basel, Switzerland; University of Basel, Basel, Switzerland; Ifakara Health Institute (IHI), P.O. Box 78373, Dar Es Salaam, Tanzania; World Health Organization Country Office, P.O Box 9292, Dar Es Salaam, Tanzania; Muhimbili University of Health and Allied Science (MUHAS), P.O. Box 65015, Dar Es Salaam, Tanzania; Centres for Health Sciences, University of the Witwatersrand, Johannesburg, South Africa; Agence de Médecine Préventive, 08BP 660, Abidjan, Côte d’Ivoire; Commission for Science and Technology (COSTECH), P.O. Box 4302, Dar Es Salaam, Tanzania

## Abstract

**Background:**

Traditionally, it has taken decades to introduce new interventions in low-income countries. Several factors account for these delays, one of which is the absence of a framework to facilitate comprehensive understanding of policy process to inform policy makers and stimulate the decision-making process. In the case of the proposed introduction of malaria vaccines in Tanzania, a specific framework for decision-making will speed up the administrative process and shorten the time until the vaccine is made available to the target population.

**Methods:**

Qualitative research was used as a basis for developing the Policy Framework. Interviews were conducted with government officials, bilateral and multilateral partners and other stakeholders in Tanzania to assess malaria treatment policy changes and to draw lessons for malaria vaccine adoption.

**Results:**

The decision-making process for adopting malaria interventions and new vaccines in general takes years, involving several processes: meetings and presentations of scientific data from different studies with consistent results, packaging and disseminating evidence and getting approval for use by the Ministry of Health and Social Welfare (MOHSW). It is influenced by contextual factors; Promoting factors include; epidemiological and intervention characteristics, country experiences of malaria treatment policy change, presentation and dissemination of evidence, coordination and harmonization of the process, use of international scientific evidence. Barriers factors includes; financial sustainability, competing health and other priorities, political will and bureaucratic procedures, costs related to the adoption and implementations of interventions, supply and distribution and professional compliance with anti-malarial drugs.

**Conclusion:**

The framework facilitates the synthesis of information in a coherent way, enabling a clearer understanding of the policy process, thereby speeding up the policy decision-making process and shortening the time for a malaria vaccine to become available.

## Background

When a decision is made to adopt and implement a new health intervention in low and middle income countries (LMICs), it often takes years or decades before the benefits of the new interventions are realized [1–5]. Thus, as new interventions become available, there is a need to improve understanding of the policy making process, as it applies to technology adoption and implementation [5–13]. The evidence-based information is needed in order to plan appropriately, set priority and choose from amongst the available alternatives [14, 15]. Lack of evidence-based information and framework could slow down the decision-making process and the process of rolling out new interventions [14, 15]. Frameworks have been useful for identifying relationships among the elements that guide and inform health policy processes [16]. In Tanzania, the decision-making process for adopting malaria interventions and new vaccines in general takes years, involving several processes before getting approval for use by the Ministry of Health and Social Welfare (MOHSW). In the case of the proposed introduction of malaria vaccines in Tanzania, a specific framework for decision-making will speed up the administrative process and shorten the time until the vaccine is made available to the target population. This study adapted a policy framework to inform policy process for introducing malaria vaccine in Tanzania, to be able to distil lessons learnt that will also serve to guide malaria vaccine policy formulation and implementation.

## Methods

### Adapting a policy framework

**Fig. 1:**
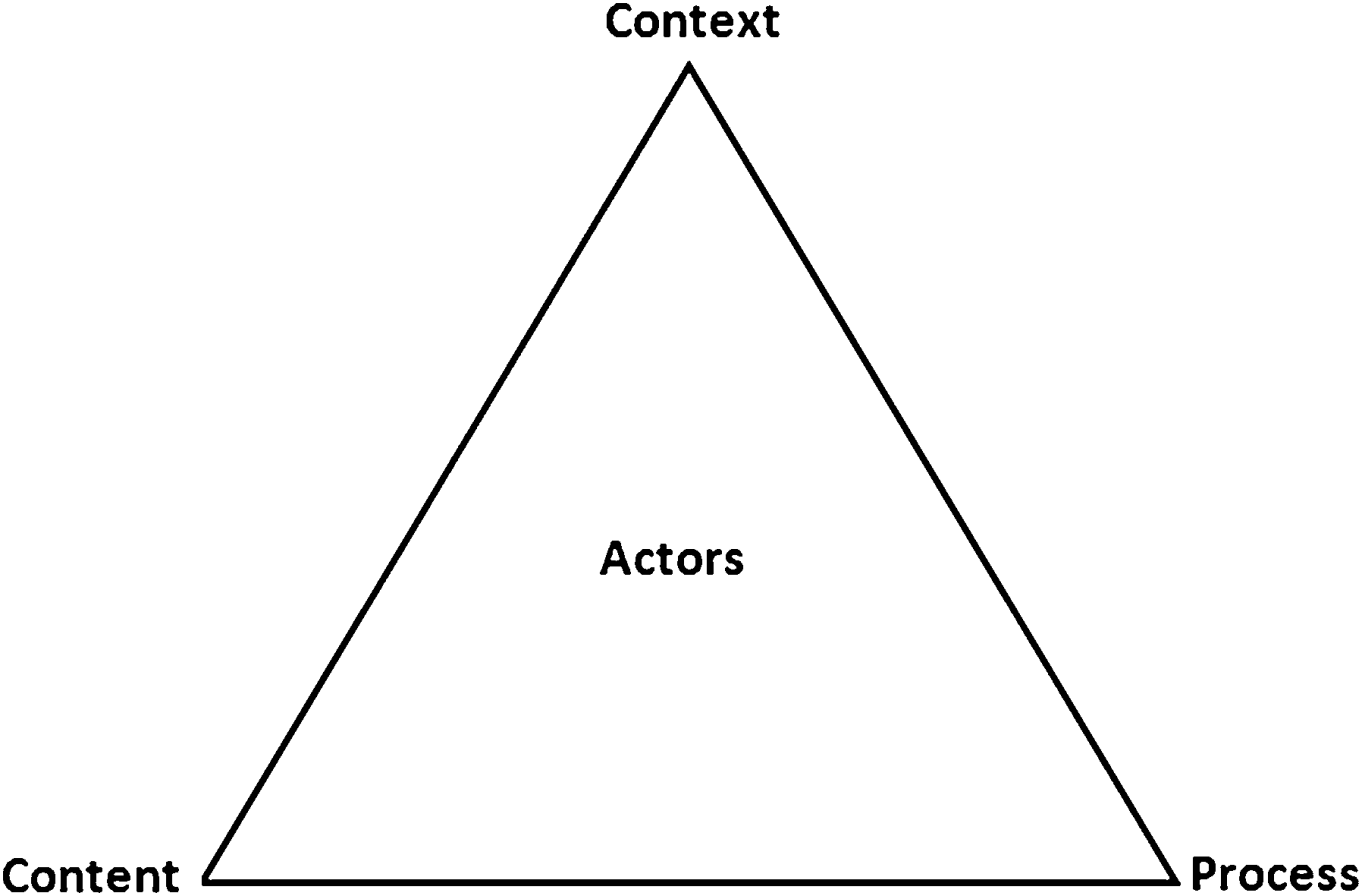
Policy Analysis Framework. Individuals, Groups, Organization

A policy framework [17] highlights ways of understanding policy processes based on four elements namely policy content, context, actors and processes involved in making and implementing policy [17] (Fig. 1). The policy framework analyses two elements; contextual (promoting and barriers factors) and process influencing the decision-making process in order to establish a mechanism that will facilitate a timely roll-out of the malaria vaccine RTS,S in Tanzania. It is important to analyse the ideal policy processes in assessing policy options for introducing new interventions and their subsequent adoption. For this case, the steps involved in the process to adopt policy decisions of malaria treatment policy change interventions in Tanzania was used. Several findings confirmed the importance of a careful assessment of the policy process which will facilitate the reform or policy change [18–20]. Also; several studies have identified the importance of barriers in policy decision making [21]. Immunization and Vaccine Development (IVD) is the key actor as malaria vaccine is expected to be delivered through IVD programme and implemented at facility level by health care providers in both private and public facilities.

### Study population

A sample of 20 key informants at the national level was assessed between July and August 2012. Participant categories included: international donors and public health stakeholders [the US Agency for International Development, the President’s Malaria Initiative, the World Health Organization (WHO), the United Nations Children fund, the Centers for Disease Control and Prevention; national and political institutions (Legislature, Members of Parliament); public health officials (Ministry of Health and Social Welfare); programme managers of the National Malaria Control Programme, the Expanded Programme on Immunization]; regulatory authorities [Tanzania Food and Drug Authority; Ministry of Finance and Economic Affairs], and professional organizations, academia and research institutions [National Institute for Medical Research, Ifakara Health Institute, African Malaria Network Trust and Tanzania Commission for Science and Technology. Purposeful sample of key informants were selected based on their knowledge and involvement in the process of changing malaria treatment policy in Tanzania. Interviews were open ended, with questions that aimed to analyse the existing policy process for new malaria interventions in Tanzania and to draw out lessons learned that could be applied to the forthcoming malaria vaccine policy adoption and implementation process.

### Data collection

The face-to-face semi-structured interviews began by soliciting verbal informed consent and permission to record the interviews. Interviews lasted between 40 and 60 min, depending on the level of detail offered by informants.

### Data analysis

Interview notes were transcribed with the aid of recordings was uploaded and imported into MAXQDA 11 software for coding based on the themes derived from the interviews related to content, context, process and actors involved in the policy process. Interviews were analysed thematically to understand the experiences of different stakeholders and to describe policy change processes. The Policy Analysis Framework was used to illustrate and interpret results.

The study protocol and interview guides were submitted and approved by the Institutional Review Board at the Ifakara Health Institute.

## Results

### Ideal policy process

In Tanzania, the process of making policy decisions for the introduction of malaria interventions involves several steps. Interviewed participants highlighted the steps as follows: (i) reviewing the available evidence from different studies with consistent results and epidemiological data; (ii) considering the availability of alternative interventions to replace the failing intervention, including the cost of the new intervention; (iii) forming a task force or technical groups composed of doctors and bilateral and multilateral partners to get additional scientific inputs and correctly package the evidence in language that can be easily understood by policy makers; (iv) getting feedback from the National Malaria Advisory Committee (NMAC), a technical body with the mandate to review technical evidence before it is made available to policy makers at the next level; (v) presenting scientific evidence to the National Malaria Control Program Manager to convince him of the need for the new intervention (NMCP is the secretariat to the NMAC). The NMCP secretariat prepares a brief summary, which, together with the recommendations from the NMAC, are presented to the Director of preventive services and to the Chief Medical Officer of the MOHSW; (vi) the Director of Preventive Services and the CMO present the findings to the MOHSW Senior Management team to get their buy-in, endorsement and approval for implementation. Normally this meeting would be held in the presence of the NMCP secretariat. The MOHSW Senior Management team comprises all Directorates (the Permanent Secretary, Director of Preventive services, Director of Curative Services, Chief Medical Officer, Director of Policy and Planning, Director of Human Resources and Director of Quality Assurance).

### Context

The analysis of the context in which malaria policy decisions are made yielded various themes. Themes were broadly categorized into one of two major areas, promoting and barriers factors.

### Promoting factors

According to interview participants, the major factors influencing the policy process for both malaria interventions:

#### Epidemiological and intervention characteristics

The WHO recommends that policy decisions for introducing new interventions be based on established evidence of the epidemiology and burden of disease and on the safety, effectiveness and efficacy of the specific intervention to prevent the target disease.

The interventions should be of high quality but the question of how MOHSW can ensure quality assurance for new interventions remains open. “*We need to set criteria for quality assurance, which we don’t have yet; the criteria to accept or not to accept the new interventions, which we do not have yet. It is an important observation you have noted”.* (“MOHSW stakeholder”).

#### Country experiences of malaria treatment policy change

“Mapping the country and looking at decisions adopted in neighbouring countries with similar settings, such as Kenya, Botswana, and Malawi, can influence policy decision outcomes”. In those countries, sulfadoxine-pyrimethamine has replaced chloroquine as the first-line drug and they have already had to revise their national drug policy guidelines, accordingly (“NMCP stakeholder”).

#### Presentation and dissemination of evidence

Technical groups translate the evidence in a manner that is digestible and understandable to policy-makers. The groups include the Medical Association of Doctors, bilateral and multilateral partners, and scientific bodies. There are lessons to be learned from past experiences. A scientific package was developed at the time that treatment policy changed from sulfadoxine-pyrimethamine to artemisinin-based combination therapy. The package included operation and orientation knowledge, an analysis of the costs and cost effectiveness of the new intervention and scientific proof that validated the intervention locally, in the field. When policy makers are well informed, they will get involved. The knowledge that the policy makers accumulate is important for adoption and approval decisions.

“*You have to simplify the language and hit the message about replacement of the intervention*” (“Bilateral & Multilateral partners stakeholders”).

“*A package of the information reflects what you need to bring as a point of reference. The Prime Minister’s Office Local Government and Regional Administration (PMOLRAG) hire and fire employees, therefore packaging information brings those employees on board and gives them a policy level of understanding*” (“Bilateral & Multilateral partners stakeholders”).

#### Coordination and harmonization of the process

Despite the increasing calls for coordination and mechanisms to improve the effectiveness of development assistance, aid remains predominantly short-term in duration, unpredictable, geographically or technologically tied, and highly fragmented. Greater harmonization and alignment of donor aid can, in conjunction with broader health financing reforms, improve the equity of health outcomes.

Significant and effective leadership is required to coordinate and harmonize the various stakeholders with stakes in the process. It will entail harmonizing the policy process, planning, resources distribution, monitoring and evaluation in collaboration with donors and other international stakeholders. The process gives the opportunity from the outset to mobilize donor funding and to demonstrate to the donors and other partners the operational and other costs related to the policy adoption and implementation and to show that the policy is cost effective. When donors and partners are taken on board at the early stages of discussing the policy change, it gives an opportunity to strategies and leverage financial and technical support towards the aim, thereby increasing the chance that the policy decision in question will be adopted (“Bilateral & Multilateral partners stakeholders”).

#### Use of international scientific evidence

The use of international scientific evidence adapted to the local context is important for informing related policy decisions. Availability of an international consultant introduces another perspective and helps to clarify scientifically proven evidence, thereby increasing the chances that a policy decision and intervention will be adopted (“Bilateral & Multilateral partners stakeholders”).

### Barriers factors

Interview respondents also identified factors that were barriers to decisions to adopt new malaria policy. These included:

#### Financial sustainability

The country cannot generate its own resources to sustainably fund new interventions from the national budget. Inadequate recurrent budgets have led to a dependency on donor funding. Sustainability of financing interventions is a challenge once when the donors withdraw their funding. For instance, there are inadequate funds for vaccine operations at national level; the government contributes 5.4 % of costs of vaccines. Specifically, the government covers the full costs of BDG, Measles, OPV and TT, while co-funding DPT-HEPB-HIB vaccine (as reported by “EPI stakeholder”).

#### Competing health and other priorities

Given its limited resources, the government must choose from among competing health priorities and other national and local priorities. Scientific evidence should justify the need for new interventions and be ranked as a priority in the MOFEA agenda:

“*It is important to understand why a particular intervention needs to be given priority, if there is treatment, prevention, larviciding, residual spraying and bed nets; all these are competing interventions, they are competing for donor funding and donors have their own interests in funding”* — (“Member of Parliament stakeholder”).

#### Political will and bureaucratic procedures

“Any new intervention takes time (two to three years) to be understood and then accepted”. Thus, planning for new interventions should start early to explore opportunities for engaging the government and donors, to take them on board, and to advocate and lobby for adoption. This is especially important in the context of government allocations for the roll out of malaria interventions and vaccines (“Bilateral & Multilateral partners stakeholders”).

#### Costs related to the adoption and implementations of interventions

All costs related to adopting and implementing interventions imply that large amounts of funds are spent on management activities rather than on actual implementation of interventions to achieve positive health outcomes (“NMCP Stakeholder”).

#### Supply and distribution

In some instances, global supply does not meet the demand for the malaria interventions, Interview respondents reported distribution issues arising from the logistics of transporting interventions from the manufacturer to the users. Other issues of concern include: whether the interventions need special transport and storage, how they are stored, availability of vehicles to facilitate transportation, and user-friendly packaging of vaccines to facilitate delivery. Another important element is training of health care personnel involved in vaccination to understand the intervention. New interventions require development and roll out of an appropriate training package for health facilities (“NMCP, Bilateral donor Stakeholders”).

#### Professional compliance with anti-malarial drugs

Access barriers related to affordability of interventions and competence of providers indicate that health infrastructure’s capacity must be increased so that clinicians comply with the recommended national policy and guidelines (“NMCP stakeholder”). These barriers are mostly influenced by: financial sustainability, competing priorities, political will and bureaucratic procedures, cost, supply and distribution and lack of compliance by users and health providers of the new malaria treatment policy.

The following list represents what stakeholders perceived were the most salient lessons learned. The potential malaria vaccine is a first generation malaria vaccine with a high probability of success at the Phase III stage; it targets specific age groups of children and is given as a consolidated package with other IVD vaccines.

- IVD has an established infrastructure which can potentially accommodate new vaccines;
- Factors for consideration are programmatic issues needed for a new vaccine, cold chain, training health workers, cost of introduction, funding opportunities available for the vaccine [Global Alliance for Vaccines and Immunization (GAVI)].

#### Key concerns from the donor group and key questions generated

- What are the operational costs of adding a new vaccine?
- What are the potential sources of funding to deliver the vaccine?
- How do you ensure supply meets demand for the vaccine?
- How do you demonstrate operational and other costs to the donor partners?

#### Package and disseminate information about the malaria vaccine

- Develop a package for the community who are users of the vaccine to let them understand exactly what the vaccine is capable of achieving. Involves trainings and use of different types of media to facilitate adverts and advocacy.

#### Lobbying and advocacy

- Any new interventions takes time (2–3 years) to be understood and then accepted, thus lobbying for the malaria vaccine should start now
- Advocacy should begin early enough as it takes time for people to understand and accept new interventions. Planning early will be important for the vaccine’s success
- Explore opportunities such as the development of new strategies (government and donors) in which to include the malaria vaccine. The vaccine should be understood as a complementary intervention to existing malaria control measures, such as insecticide-treated nets, artemisinin-based combination therapy and diagnostics
- Integrate malaria vaccine with other opportunities, such as child health day, malaria campaigns in general, and use of advocacy avenues.
- To secure enough funding and involve other stakeholders.

#### Planning, financing and implementation

- There should be adequate analysis of the vaccine system in line with the introduction of the forthcoming malaria vaccine (storage, delivery, and packaging).

#### Integration and complementarity

- Attention should be paid to the documents or guidelines to show how the vaccine relates to and complements other ongoing malaria interventions.
- Consider options for delivery at primary levels using existing interventions, e.g. the delivery of a booster dose should be explored and documented in the implementation guidelines.

#### Involvement of front line implementers

- Sensitization of health workers has to begin early enough to improve on motivation and any negativity projected from them to community.
- Involvement of health workers can be done through several, small gestures. For example, holding meetings between Council Health Management Teams (CHMTs) and health workers when they conduct supervision, informing and advising them to accept a new vaccine.

#### Continuity and sustainability

- Have a clear plan of what the funding sources would be after GAVI support ends
- Use opportunity to develop new health-related strategies to include the vaccine so that it is considered in funding
- Ensure that there will be enough production so that procurement will not be affected by low production.

## Discussion

The framework method has been developed and used widely in many countries and to address a variety of health policy concerns [22]. The policy framework combines the concepts of content, context, actors and process to understand the policy process and to plan for effective implementation of interventions [17]. A policy framework in this study built on the literature and was based on experiences and observations of the policy process and the factors influencing policy decisions in Tanzania. It was used to organize information in a way that explains the drivers of policy change and to gauge understanding and lessons learnt from the introduction of new malaria interventions through policy change.

The framework is feasible and can be used in the Tanzanian context. Although Tanzania has not yet introduced a malaria vaccine, the framework contributes to understanding a very complex and highly political subject—policy analysis. It assists in unpacking the national level discussion, involving evidence-based information, stakeholders’ interactions and political commitment; factors that are all important for planning the forthcoming malaria vaccine. Promoting factors, such as safety and efficacy, WHO protocol and decisions adopted in other countries, such as Burkina Faso [23, 24], Ghana [25], Gabon [26], Malawi [27] and Kenya [28]. Harmonization of the policy process across departments and collaboration between policy makers and scientists were identified in Burkina Faso [23] and Malawi [27]. The importance of technical assistance from the WHO and other interested donors was also identified in Burkina Faso [23], Gabon [26], Malawi [27] and Kenya [28]. Among the identified barriers were the lack of sustainable financing in Burkina Faso [24], Gabon [26] and Kenya [28]; and non-adherence to treatment in Burkina Faso [24], Mozambique [29] and Kenya [28]. Effective communication supporting the correct use of medicines can counteract non-adherence and use of in-effective medicines [30, 31]. It can also be done through engaging the private sector and encouraging hospitals and pharmacies to adhere to national guidelines [23].

### Strength and limitation

The framework described the potential for introducing a malaria vaccine in the health system while critically observing policy formulation and implementation. The framework approach has its limitations. It highlights some information while minimizing or excluding others [32], in order to focus on aspects that are relevant to the study context [33]. The framework may or may not be applicable to other low-income countries with similar contexts. Its applicability depends on whether the policy is appropriate to the needs of a specific country and is feasible in a low-resource settings [34].

## Conclusion and recommendations

The framework is used at the national level, while overall policy recommendation of RTS,S vaccine is made at the global level. The framework can be used once the global policy recommendation is made. The framework is a comprehensive tool that enables one to unpack the factors surrounding the decision to introduce a potential malaria vaccine in Tanzania. Furthermore, the framework provides an effective way to improve knowledge of the policy process and to inform the policy decision-making process for new malaria interventions, generally, and for a forthcoming malaria vaccine, specifically. Lastly, the framework facilitates the synthesis of information in a coherent way, enabling a clearer understanding of the policy process, thereby speeding up the policy decision-making process and shortening the time until the forthcoming malaria vaccine becomes available. The framework is useful and applicable, still further validation is required.

The framework is appropriate and recommended for various settings depending on the steps involved in decision-making process and availability of contextual (promoting and barriers factors) information to inform the policy decision process, which also implies a need of study to understand those factors before a policy decision is made on RTS,S adoption. It can be useful as a step in the direction of research that supports better formulation and implementation of malaria interventions policies in Africa.

## Abbreviations

CHMTs: Council Health Management Teams
IVD: Immunization and Vaccine Development
LIMCs: low and middle Income Countries
MOFEA: Ministry of Finance and Economic Affairs
MOHSW: Ministry of Health and Social Welfare
NIMR: National Institute for Medical Research
NMAC: National Malaria Advisory Committee
NMCP: National Malaria Control Programme
PMI: Presidents Malaria Initiative
PMOLRAG: Prime Minister’s Office Local Government and Regional Administration
TFDA: Tanzania Food and Drug Authority
WHO: World Health Organization
GAVI: Global Alliance for Vaccines and Immunization
